# Repetition suppression/enhancement effects as a result of attractor dynamics in local cortical networks

**DOI:** 10.1186/1471-2202-14-S1-P277

**Published:** 2013-07-08

**Authors:** Elisa M Tartaglia, Nicolas Brunel, Gianluigi Mongillo

**Affiliations:** 1 Center for Neuroscience and Cognitive Systems @UniTn, Istituto Italiano di Tecnologia, Rovereto, Italy; 2 Departments of Statistics and Neurobiology, University of Chicago, Chicago, IL, 606037, USA; 3 Laboratoire de Neurophysique et Physiologie, CNRS UMR 8119, Université Paris Descartes, 75270 Paris, France

## 

Electrophysiological studies on short-term memory tasks have consistently reported three neural effects in associative cortices (i.e. ITC, PFC and PPC; [1]): a modulation of neuronal responses following stimulus repetition (repetition suppression --RS-- and repetition enhancement --RE-- effects) as well as sustained neural activity after the first stimulus has disappeared (persistent activity --PA--). For instance, in a standard delayed match-to-sample task, two visual stimuli are presented one after the other, with a delay period separating the two presentations. The task consists in recognizing if the first stimulus (the "sample") matches the second ("match") or not ("non-match"). While a certain proportion of cells respond less to a match stimulus than when the same identical stimulus is presented as sample or non-match (RS), some others show the opposite behavior, with an enhanced response to the match rather than to the sample or to the non-match (RE). Sustained neural activity (PA) is commonly observed in the delay period separating the presentation of sample and match/non-match. Although consistently observed in the same cortical areas and, often, in the same cell (e.g. a cell which shows RE also shows PA), these effects have never been related in terms of cellular and/or network mechanisms.

Here, we argue that repetition effects, as well as persistent activity, are a signature of attractor dynamics in local networks. We show, by using a model network of spiking neurons which implements attractor dynamics, that the current network state affects the response to incoming inputs: persistent activity i.e. the internal dynamic state of the network, interacts with the incoming stimulus suppressing the responses of those neurons which are not highly selective for that stimulus (RS). On the other hand, the responses of those neurons which respond optimally for the incoming stimulus tend to be enhanced (RE). In agreement with the experimental data, we find that the less selective neural population shows a response to the match which is suppressed with respect to both sample and non-match. To the opposite, the most selective neurons'response to the match is enhanced with respect to both sample and non-match [2]. Moreover, the proportion of cells showing RE is correlated with the proportion of cells showing PA, consistently with the experimental data [3]. Interestingly, the magnitude of RE and RS effects should increase, according to the model predictions, with increasing firing rate during PA.

Existing models and theories of repetition effects (e.g. [4]) suggest that suppression and/or enhancement of the neural response might be due to a modulatory top-down signal. However, the short latency observed in repetition effects implies that the modulation of the response should rather occur within the same cortical area. Here, we can explain in a unique and parsimonious framework the three main correlates of short-term memory and, more importantly, we can do so by fully relying on the local recurrent circuitry.
